# The Impacts of Family Educational Investment on Mental Health of Chinese Parents: Mediating and Moderating Effects

**DOI:** 10.3389/ijph.2023.1605566

**Published:** 2023-06-02

**Authors:** Ping Zhang, Zhewei Xu

**Affiliations:** China Center for Special Economic Zone Research, Shenzhen University, Shenzhen, Guangdong, China

**Keywords:** family educational investment, parental mental health, social integration, social participation, workload, measures

## Abstract

**Objectives:** This review aims to explore the impact of family educational investment on parents’ mental health in China. Through this study, we reveal the current public health challenges and propose some solutions.

**Methods:** Family educational investment takes three forms: economic investment, emotional investment, and time investment. This study examined the mediating effect of social integration and the moderating effect of social participation and workload on the relationship between family educational investment and parental mental health.

**Results:** Economic investment, emotional investment and time investment were all negatively correlated with parental mental health. Social integration could better explain the detrimental effects of family educational investment on parental mental health, and that social participation and workload could play a significant negative and positive moderating role, respectively.

**Conclusion:** Family educational investment, particularly emotional investment, plays an important and negative role in influencing parental mental health. To cope with the increased pressure brought about by educational competition, the state, society, and individuals all need to take measures.

## Introduction

**FIGURE 1:**
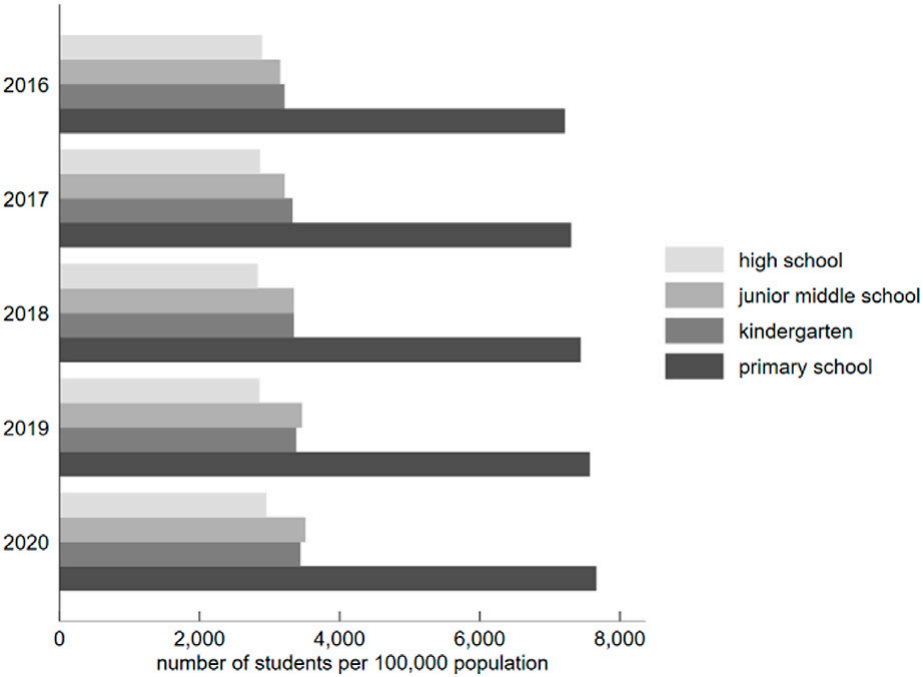
Changes in the number of students at different stages of study from 2016 to 2020 (China. 2016–2020) Source: China’s National Bureau of Statistics (http://www.stats.gov.cn).

With the fast expansion of the global economy, education has seen unparalleled growth around the world. Figure 1 depicts the trend of a constant rise in the proportion of students in all stages of education per 100,000 people in China from 2016 to 2020, which represents the flourishing development of education in China. However, as a result of the imbalance of educational resources, the increasing difficulty of entering a good college, and the sharp increase in employment pressure, education competition in China has intensified. Chinese parents are likewise becoming more eager to invest in their children’s education, and the resultant educational pressure is an important factor affecting parental mental health. From the perspective of the state and society, parental mental health has always been a hot topic because it plays an indispensable role in children’s education and growth. Therefore, studying the influence of the educational pressure behind family educational investment on parental mental health is of great practical importance. Existing research focuses on explicit investment behavior (e.g., economic investment) in family educational investment. However, implicit investment (e.g., psychological investment and time investment) receives considerably less attention in households. Moreover, although some studies have focused on the impact of family educational investment on children’s mental health and academic performance [1], they have rarely addressed the influence and mechanism of educational stress on parents’ mental health. This paper aims to further discuss and research these issues.

Education is a crucial endogenous mechanism of human capital accumulation, which is an essential reason why parents focus on education. Studies have shown that a well-educated person who attends an elite university has higher income and better career prospects [2], as well as an increased likelihood of accumulating social and academic capital [3]. In China, the Confucian cultural tradition of “Education changes destiny” has always attached great importance to children’s education, which directly determines how much parents invest in family education. Given the rise of increasingly equitable school education, the center of competition has gradually shifted from school education to shadow education (i.e., after-school tutoring) in order to maintain educational inequality among families of different classes [4]. Thus, shadow education has become an additional way for parents to improve their children’s academic skills, whose high cost and diverse choices bring more challenges to family educational investment and increase parental educational pressure.

Empirical studies show that social integration, social participation, and work intensity are all important factors affecting parents’ mental health [5–8]. Social integration and social participation reflect the social networks and social interaction between people. In comparison to social participation, social integration stresses subjective emotional integration and encompasses a broader range of activities. Individuals who have a high sense of social integration and social participation can easily obtain social support and then effectively recover their mental health [9–12]. As for work intensity, scholars are more interested in the topic of overwork. Studies have shown that overwork takes a toll on physical health and crowds out social participation [13, 14], leading to negative effects on mental health. Therefore, considering previous research on this topic, we suspect that the above three factors may play either a mediating or moderating role in the influence of parental educational stress on parental mental health.

**FIGURE 2:**
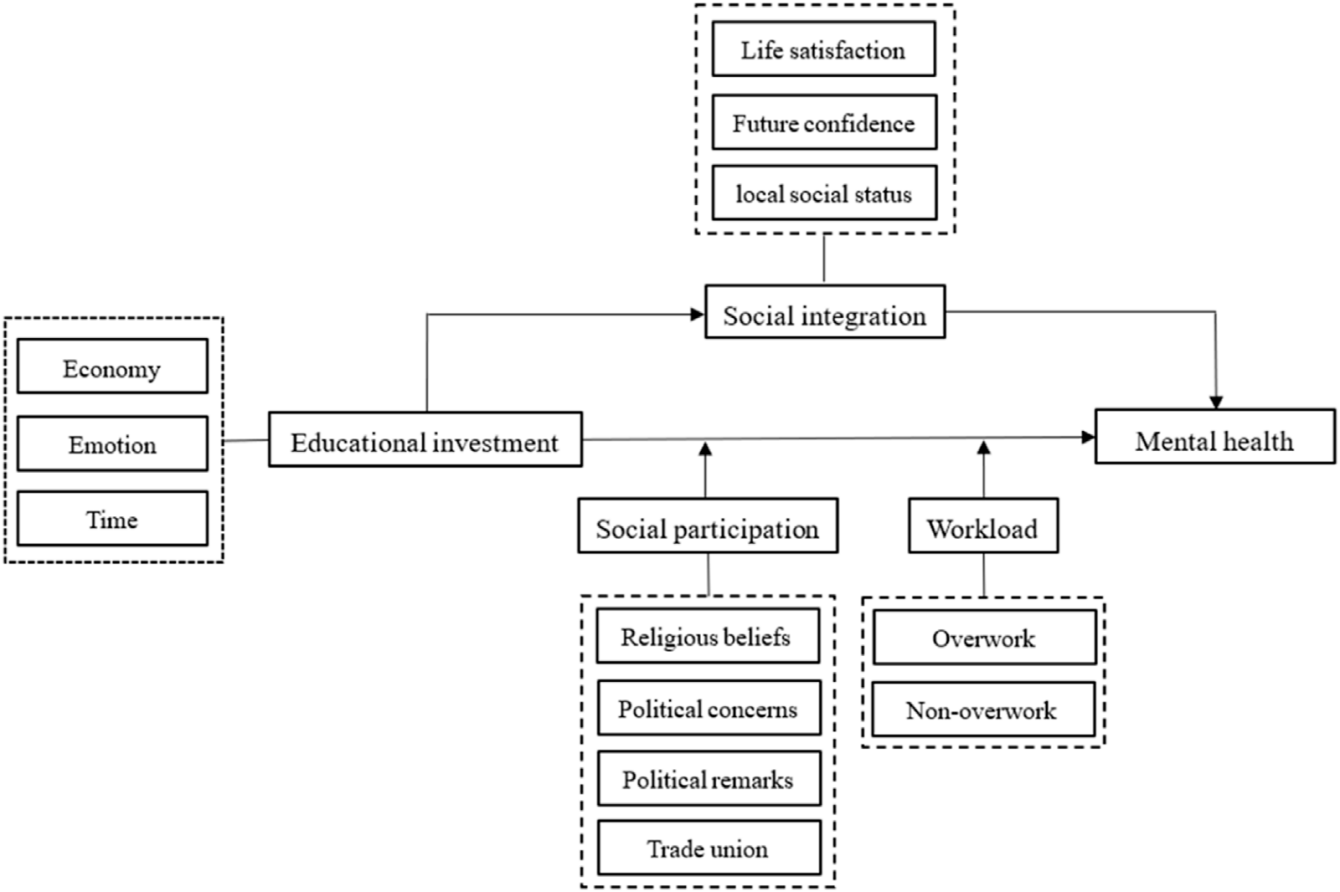
The theoretical framework of family educational investment and parental mental health (China. 2023).

The goal of this study is to investigate the impact of parental educational stress on their mental health, as well as the potential effect mechanisms. Given the scope of the available data, we divided family educational investment (i.e., parental education pressure) into three aspects: economy, emotion, and time. Research has found that all three aspects are negatively correlated with parental mental health. As an intermediary factor, social integration can significantly explain the negative effects of family educational investment on parental mental health. Furthermore, social participation significantly attenuates the negative effect of economic investment on parental mental health, while workload significantly amplifies the negative effect of emotional investment on parental mental health. The theoretical framework of this study is shown in Figure 2.

## Methods

### Data and Sample

This study used data from the 2018 China Family Panel Studies (CFPS 2018, https://opendata.pku.edu.cn). CFPS 2018 is a national comprehensive family social tracking survey carried out by the China Center for Social Science Surveys at Peking University. CFPS 2018 mainly includes three dimensions: individuals, families, and communities, involving economic behaviour, education achievements, family relationships and dynamics, population migration, physical and mental health, and many other research topics. The sample covered 25 provinces (municipalities and autonomous regions) in China, with interviews conducted with 15,000 households and about 44,000 individual questionnaires collected, representing 95% of China’s population. Among the respondents, there were many baseline gene members who are permanently tracked by the survey as family members related to the family by blood/marriage/adoption as defined by the 2010 baseline survey. Additionally, future biological/adopted children of these genetic members are also considered genetic members and will be permanently tracked. The completion rate of baseline gene members in CFPS2018 was 64.5%, which was in line with international standards when compared to the UK Household Tracking Survey (UKHLS) conducted at the same time.

Data processing in this study is mainly divided into three steps. In step 1: combine the personal self-response questionnaire with the children’s response questionnaire according to the personal ID code. The personal self-response questionnaire mainly records the personal situation of parents, while the children’s response questionnaire mainly records the basic situation of their children. The number of children can match the family ID code obtained from the family member questionnaire. In step 2, given that the primary family educational investment takes place before the child reaches adulthood, we decided to sample parents whose children were currently in pre-college education. In step 3: remove data with missing information or extreme values. After data processing, the data of 4,078 respondents were used in this study.

### Variables

#### Mental Health

Mental health reflects a person’s inner life and state. Prior studies have mainly measured mental health in two ways. One is self-rated mental health (SRMH), which requires respondents to rate their overall mental health on one item on a five-point scale from excellent to poor [15]. The other is to assess only one or more aspects of mental health, such as anxiety, stress, and depression [16, 17]. In this study, we used CESD20sc data from CFPS 2018 to measure the mental health of parents; this scale specifically measures the level of depression. It was computed by equipercentile equating based on the Center for Epidemiologic Studies Depression Scale (CES-D). Higher scores indicate more depression. To make the data more intuitive, we defined mental health as the reciprocal of CDSE20sc multiplied by 100, such that a higher value now indicates better mental health.

#### Educational Investment

Family educational investment is an important means of human capital accumulation [18]. Children’s education has always been an extremely worrying topic for parents, whether out of concern for their children’s success or the desire to be cared for in old age. In this study, we divided family educational investment into three parts: economic investment, emotional investment, and time investment. Economic investment was measured by (ln) total household spending on children’s education over the past year divided by the household income level, with household income level ranked from 1 = *low income* to 4 = *high income*. The higher the quotient, the greater the pressure of economic investment. Emotional investment primarily assessed how much psychological care and involvement parents have in their children's schooling, which was measured based on a combination of whether the child attended tutoring classes or had private tutors, the frequency of parents giving up watching television for their children's learning (numerical standardization), and the frequency of stopping a child from watching television (numerical standardization). The higher the overall value, the more importance parents attached to their children's studies and the greater the emotional pressure. Time investment referred to the time parents spent personally tutoring their children, as measured by the number of hours of tutoring per week. All three parts of family educational investment took into account the number of children in a family. Economic investment and time investment were summed up according to the number of children in the family, while emotional investment was averaged according to the number of children in the family.

#### Mediating and Moderating Variables

Social integration was chosen as the mediating variable in this study. Several previous studies have shown that social integration is closely related to mental health [9, 19]. Following the example of Chen and Hu [20], life satisfaction, future confidence, and local social status were assigned scores of 1–5 respectively to measure social integration. The higher the overall score, the higher the degree of social integration. The moderating variables selected for this study were social participation and workload. Social participation included four questions: whether you had religious beliefs, whether you were informed about politics through either TV or the Internet, whether you made political remarks through the Internet, and whether you were a member of a trade union. An affirmative answer to any of these was taken to indicate the presence of social participation, and a value of 1 was assigned for the variable. Workload was measured by whether the participant was overworked or not. The judgment standard was based on the Labor Law of the People’s Republic of China. If workload exceeded 44 h per week, it was defined as overwork, and a value of 1 was assigned.

#### Control Variables

Regional economic and demographic factors were used as control variables. These were: Gini coefficient (continuous variable), such that the higher the value, the more unbalanced the regional economy; age (continuous variable); gender (categorical variable); marital status (categorical variable); work status (categorical variable); educational level (continuous variable); (ln) social capital (continuous variable), which was measured as the natural logarithm of the amount of renqing consumption in the past year because renqing consumption is a necessary expense for maintaining personal relationships in China; community trust (continuous variable), expressed in terms of trust in neighbors; (ln) household net assets (continuous variable); weekly physical exercise (continuous variable); and self-rated physical health (continuous variable), ranked from 1 = *unhealthy* to 5 = *very healthy*.

#### Methods Description

Considering the dependent variable of mental health was a continuous variable, a series of OLS regression models were conducted to estimate the effects of family educational investment on parental mental health. Firstly, we evaluated the effects of economic investment, emotional investment, and time investment on parental mental health in a basic regression model. Next, we construct the mediating regression model and the moderating regression model. In analyzing the mediating effect, we used the two-stage method to test the mediation hypothesis. In this study, the mediating variable was social integration. We tested the influence paths from family educational investment (economic investment, emotional investment, and time investment, respectively) to social integration to mental health. In the moderating analysis, we used interaction terms to measure the moderating effects of social participation and workload. The basic regression model is stated in Eq. 1, the mediating regression model and the moderating regression model are stated in Eqs 2–4, respectively:

$$y_{i}=\alpha +\beta_{1}x_{1i}+\beta_{2}x_{2i}+\beta_{3}x_{3i}+X_{i}\gamma +\varepsilon_{i}$$ (1)

$$m_{i}=\gamma_{0}+\rho_{1}x_{1i}+\rho_{2}x_{2i}+\rho_{3}x_{3i}+X_{i}\delta +\varepsilon_{i}$$ (2)

$$y_{i}=\alpha +\beta_{1}x_{1i}+\beta_{2}x_{2i}+\beta_{3}x_{3i}+\mu m_{i}+X_{i}\gamma +\varepsilon_{i}$$ (3)

$$y_{i}=\alpha +\beta_{1}x_{1i}+\beta_{2}x_{2i}+\beta_{3}x_{3i}+\rho_{1}x_{1i}\omega_{i}+\rho_{2}x_{2i}\omega_{i}+\rho_{3}x_{3i}\omega_{i}+\varphi_{1}x_{1i}\tau_{i}+\varphi_{2}x_{2i}\tau_{i}+\varphi_{3}x_{3i}\tau_{i}+X_{i}\gamma +\varepsilon_{i}$$ (4)

where $y_{i}$ is the estimation of dependent variables (parental mental health); $x_{1i}$ , $x_{2i}$ and $x_{3i}$ are the estimation of independent variables (economic investment, emotional investment, and time investment, respectively); $m_{i}$ is the estimation of mediating variables (social integration); $\omega_{i}$ and $\tau_{i}$ are the estimation of moderating variables (social participation and workload, respectively); $X_{i}$ are the control variables; and $\varepsilon_{i}$ is the perturbation term.

In this study, we have a large number of independent variables. In order to verify the validity of the model, we calculated the variance inflation factor (VIF) to analyze the collinearity in each regression model. The result shows the mean VIF is 1.09. And the VIFs of variables are always less than 10, indicating that there is no collinearity in our models. The model is proved valid.

## Results

### Descriptive Statistics of the Variables

Supplementary Table S1 presents the descriptive statistics of all the variables used in the analysis. It can be observed that the average mental health level of respondents, based on the total sample (1.39–4.55), was high and stood at 3.19. Similarly, the levels of social integration and social participation were also high. The average regional Gini coefficient was 0.4, suggesting that the regional economic imbalance still persists in China, and it is alarming internationally. Most of the respondents were married (97.43%), and more than half of them were non-farm employees in terms of working status. The average educational level of the respondents was low, standing at 1.79. However, the average self-rated health score of the respondents was 3.22, implying that most respondents were healthy.

### Basic Regression

Supplementary Table S2 displays the results of the regression conducted on family educational investment and parental mental health. Model 1 indicates that family educational investment was significantly and negatively related to parental mental health. Specifically, the three variables, economic investment, emotional investment, and time investment were each significantly and negatively associated with the respondents’ mental health (coefficient =−0.012 , p<0.05 ; coefficient =−0.036 , p<0.05 ; coefficient =−0.002,p<0.1 ; respectively).

### Robustness Test

In order to test the robustness of the regression results, we referred to authoritative literature [21] and adopt the method of replacing the explained variable to test the robustness and effectiveness of the basic regression model. According to our data source (CFPS2018), the psychological status of the respondents is reflected through the Center for Epidemiologic Studies Depression Scale (CES-D). The Depression Scale provides us with two indicators: CESD8 and CESD20sc. Compared to CESD20sc, CESD8 does not have cross-year comparability. For example, for the same respondent, their 2016 CESD8 score is not comparable to their 2018 CESD8 score. However, considering that the sample data in this study is cross-sectional data, the CESD8 score can also be used to measure the psychological health of respondents in this study context. Therefore, this paper uses CESD8 to replace CESD20sc in measuring the mental health level of respondents to verify the robustness of the regression results. At this time, the regression result is still significant, indicating that family educational investment was significantly and negatively related to parental mental health. The research hypothesis of this paper has been verified once again. The results are shown in Supplementary Table S2 Model 2.

### Heterogeneity Test

Model 3 and Model 4 were based on grouped regression according to Gini coefficient = 0.4 (which was chosen because it is the international warning line). Model 3 represents a relatively balanced economy (Gini coefficient ≤ 0.4). In this model, emotional investment and time investment both had a significantly negative correlation with mental health, while economic investment had no significant influence. By contrast, Model 4 represents an imbalanced economy (Gini coefficient > 0.4), indicating more competition in economic investment for children’s education in economically unbalanced areas. Model 5 and Model 6 were based on grouped regression according to education = 2. Model 5 represents a situation in which the respondents’ educational level was junior high school or below (education ≤ 2). It shows that economic investment had a significantly negative impact on respondents’ mental health. Model 6 represents a situation in which the respondents’ educational level was above junior high school (education > 2). In this model, the negative effects of economic investment and time investment were more significant.

### Mechanism: Mediating and Moderating Effects

Supplementary Table S3 displays the regression results for the relationship between family educational investment and parental mental health with the mediating effect of social integration. The dependent variable in models 7, 9, 10, 12, 13, and 15 was mental health, while the dependent variable in models 8, 11, and 14 was social integration. Models 7 and 8 demonstrate that economic investment was significantly and negatively related to respondents’ mental health and social integration (coefficient =−0.012 , p<0.05 ; coefficient =−0.035 , p<0.05 ; respectively). Models 10 and 11 indicate that emotional investment was significantly and negatively related to respondents’ mental health and social integration (coefficient =−0.041 , p<0.05 ; coefficient =−0.106 , p<0.1 ; respectively). Models 13 and 14 show that time investment was significantly and negatively related to respondents’ mental health and social integration (coefficient =−0.003 , p<0.05 ; coefficient =−0.008 , p<0.1 ; respectively).

**FIGURE 3:**
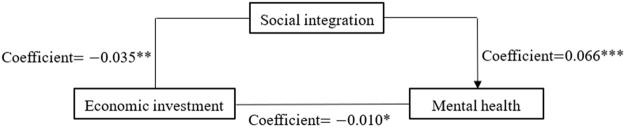
Coefficients for economic investment→social integration→mental health (China. 2018).

**FIGURE 4:**
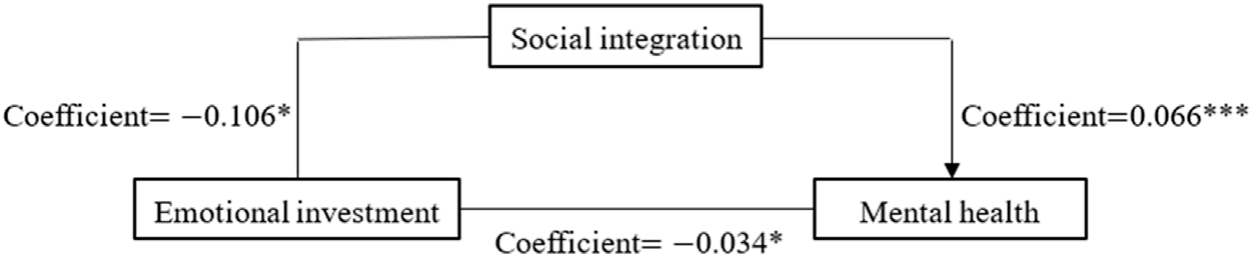
Coefficients for emotional investment→social integration→mental health (China. 2018).

**FIGURE 5:**
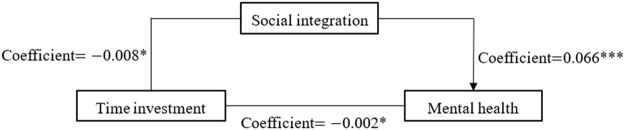
Coefficients for time investment→social integration→mental health (China. 2018).

To reflect the regression results in Supplementary Table S3 more intuitively, we portray the three paths of mediating effects in Figures 3–5, respectively. Figure 3 shows the mediating effect of social integration on the relationship between economic investment and mental health (direct effect =−0.010 , indirect effect =−0.002 , proportion of mediation effect =19.25% ). Figure 4 shows the mediating effect of social integration on the relationship between emotional investment and mental health (direct effect =−0.034 , indirect effect =−0.007 , proportion of mediation effect =17.07% ). Figure 5 shows the mediating effect of social integration on the relationship between time investment and mental health (direct effect =−0.002 , indirect effect =−0.001 , proportion of mediation effect = 33.33% ).

Supplementary Table S4 presents the regression results for the moderating effects of social participation and workload on the relationship between family educational investment and parental mental health. The results of model 16 show that economic investment, emotional investment, and time investment all had significant negative effects on mental health. The interaction term regression results of model 17 reveal that social participation weakened the negative relationship between economic investment and mental health while workload amplified the negative relationship between emotional investment and mental health.

## Discussion

Parental mental health is essential for a happy family. However, in China, parents have been struggling with their children’s education for a long time, and little is known about how education affects their mental health. In this paper, we examined the effects of family educational investment on Chinese parents’ mental health from three perspectives, using data from CFPS2018 across 4,078 respondents sampled in China. Based on the personal perspective of the respondents in the sample, we conducted heterogeneity analysis and mechanism analysis.

In the basic regression model, we found that family educational investment was significantly and negatively correlated to parental mental health, indicating that parental educational pressure behind family educational investment is harmful to their mental health. This result is in line with literature discussing parental burnout and the motherhood dilemma [22–24]. Parents’ parenting style, peer effect, children’s cooperation, and other factors also influence parents’ perceptions of pressure. In China, “Tiger mother-style parenting” is a typical feature of family education [25]. To make children do well in school and life, parents using “Tiger mother-style parenting” need to be strictly self-disciplined [26, 27], which makes them bear heavy burdens. With the phenomenon of “education involution” in the form of private tutoring becoming increasingly significant, it inevitably intensifies the peer effect of educational competition, which is particularly evident on social networks where information is transparent. Making social comparisons on social networking sites has been linked to higher levels of depression [28]. Moreover, children in the rebellious stage tend to disobey their parents’ discipline, which undoubtedly intensifies the psychological burden on their parents. The continuous influence of these factors on parental education pressure gives rise to societal phenomena such as parental burnout and the motherhood dilemma, which seriously affect parents’ enthusiasm for parenting and personal career planning, and are not conducive to parental mental health.

In the heterogeneity analysis model, we found that parents in economically unbalanced areas faced greater pressure of economic investment but less pressure of psychological investment and time investment, while parents in economically balanced areas were the opposite. This indicates that there is a disparity between the rich and the poor in regions with uneven economic development, and parents with average income levels frequently pay more for their children’s education. In addition, parents who have higher education levels, economic investment and time investment have a greater impact on their mental health than emotional investment. One possible reason for this is that parents with higher education may make more after-school investments for their children and invest more in the tutoring process. Generally speaking, parents with higher education will enjoy more economic rewards. When they spend more time tutoring their children, they would lose more economic rewards for the same amount of tutoring time due to opportunity cost, which puts a heavy psychological burden on them.

Furthermore, in the mechanism model, we discovered that social integration can explain the detrimental effects of family educational investment on parental mental health, and social participation and workload can play a significant negative and positive moderating role respectively. This is consistent with existing studies [9–11], whose conclusions highlight the function of social networks as a link between social integration and mental health for individuals. Generally speaking, social networks (such as family and friends networks, neighbors networks, and social organizations) can affect a person’s mental health through social support, resource-sharing, and emotional attachment [29, 30]. The higher the level of social integration or social participation of individuals, the more developed the social networks are. This makes it easier for individuals to obtain social support, resources, and emotional attachment, thus strengthening their psychological defense line.

To the best of our knowledge, the present work contributes to the current literature mainly in three ways. Firstly, we used a large sample in our study. Secondly, we split family educational investment into three categories: economic investment, emotional investment, and time investment, which have not been covered in other literature. Thirdly, our conclusions have strong practical significance and can provide a reference for policymakers and practitioners.

There are two limitations that should be acknowledged when considering our results. Firstly, due to limited data availability, this paper did not conduct a comparative analysis of parental mental health between the local population and the floating population. However, in migrant cities, differences in the educational experiences of children from these two populations exist due to the hukou system (i.e., the household registration system) [31]. Secondly, due to limitations of the questionnaire survey, although we collected as many factors as possible reflecting psychological investment, the characterization of emotional investment was not comprehensive enough. These should be improved in future studies.

**FIGURE 6:**
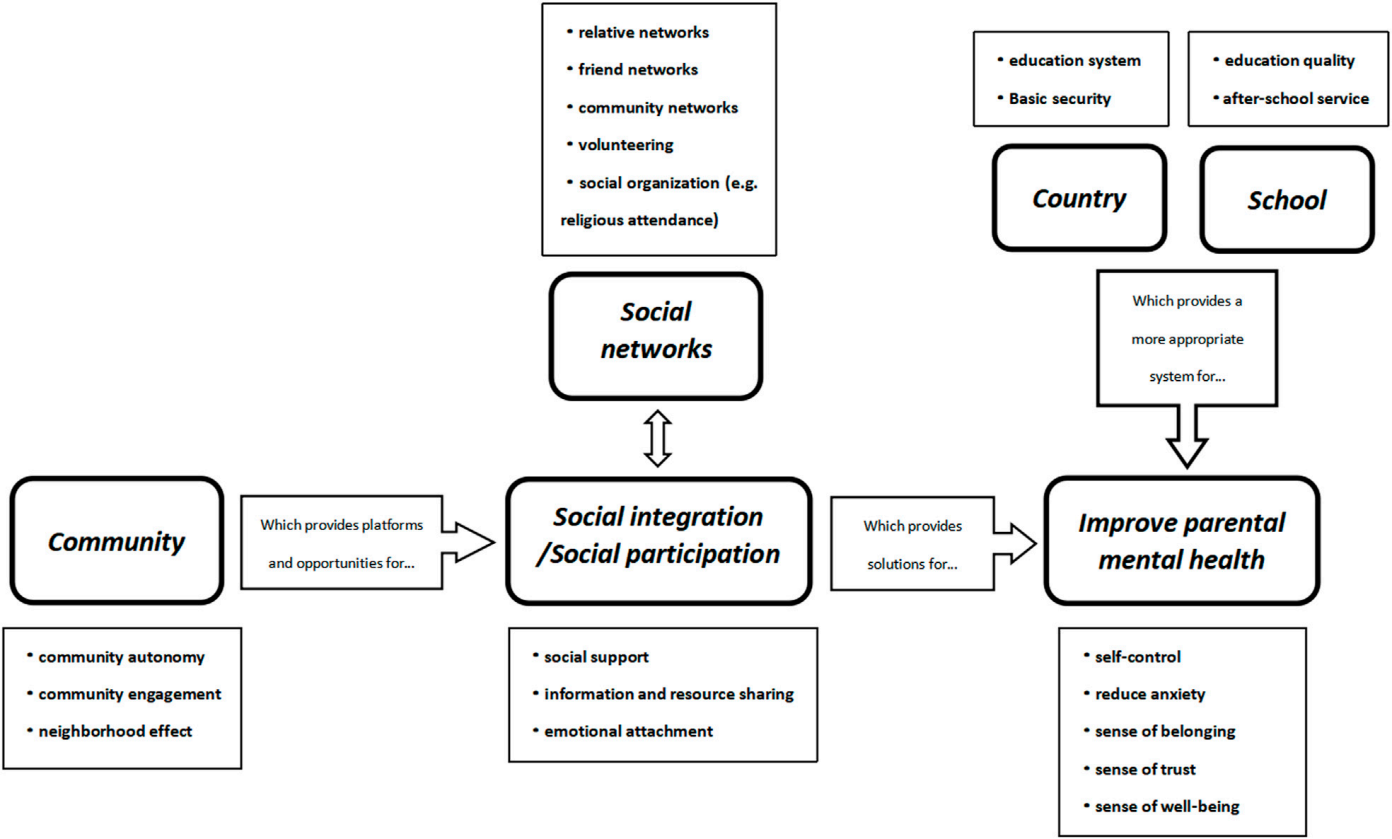
Policy recommendation framework (from national, social, and individual perspective) (China. 2023).

Even with these limitations, our findings offer novel evidence supporting the idea that many aspects of family educational investment can be detrimental to parents’ mental health. From a policy perspective, firstly, Chinese governmental agencies should promote and advance quality-focused education, and place emphasis on thorough assessment and multiple admissions. Additionally, the government should further improve basic security systems such as employment, healthcare, and social security, providing more protection for parents’ mental health. Secondly, schools should systematically improve school education quality, addressing inadequate and unbalanced distribution of high-quality educational resources. Communities should urge people to participate in grassroots governance and community collective activities, which widen parents’ social networks and provide more social support for them. Thirdly, individuals should actively participate in activities held by the community or other social organizations, gaining a broader sense of belonging, trust, and happiness, then reducing the psychological burden brought about by the pressure of their children’s education. The specific policy recommendation framework is shown in Figure 6.

### Conclusion

This study found that the three aspects of family educational investment, i.e., economic investment, emotional investment, and time investment, have significant negative impacts on parental mental health. Social integration plays a significant mediating role in the relationship between family educational investment and parental mental health. Social participation attenuates the negative effects of economic investment on parental mental health, while workload amplifies the negative effect of emotional investment on parental mental health. This study shows that under the background of increasingly intense educational competition, family educational investment is an important factor affecting parental mental health, which is a serious issue worthy of attention from both the country and society. Our research advocates strengthening the role of communities and encouraging individuals to actively participate in social activities.
