# Supplementary material for: The Impacts of Family Educational Investment on Mental Health of Chinese Parents: Mediating and Moderating Effects
Source: Int J Public Health. 2023 Jun 2;68:1605566. doi: 10.3389/ijph.2023.1605566 (PMC10272405; doi:10.3389/ijph.2023.1605566)
Supplement: Supplementary file 1 [file DataSheet1.docx]

### The Impacts of Family Educational Investment on Mental Health of Chinese Parents: Mediating and Moderating Effects

Supplementary File

Table S1 Descriptive statistics of the variables used in the analysis (n=4078) (China. 2018)

| Variables | Mean (SD)/percentage |
| --- | --- |
| *Mental health* | 3.19 (SD=0.66) |
| *Economic investment* | 4.14 (SD=2.38) |
| *Emotional investment* | 1.03 (SD=0.55) |
| *Time investment* | 5.36 (SD=7.27) |
| *Social integration (0-15)* | 11.15 (SD=2.07) |
| *Social participation* | 0.88 (SD=0.33) |
| *Workload* | 0.62 (SD=0.49) |
| *Gini coefficient* | 0.40 (SD=0.03) |
| *Age* | 37.36 (SD=6.61) |
| *Gender (%)* |  |
| *Male* | 52.97% |
| *Female* | 47.03% |
| *Marital status (%)* |  |
| *Married* | 97.43% |
| *Cohabit* | 0.17% |
| *Single* | 0.12% |
| *Divorced* | 1.81% |
| *Widowed* | 0.47% |
| *Work status (%)* |  |
| *Farm self-employment* | 28.57% |
| *Non-farm self-employment* | 15.62% |
| *Farm employed* | 2.48% |
| *Non-farm employed* | 53.33% |
| *Educational level (0-6)* | 1.79 (SD=1.35) |
| *Social capital* | 7.57 (SD=1.98) |
| *Community trust (0-10)* | 6.71 (SD=1.98) |
| *Net asset* | 12.15 (SD=3.12) |
| *Physical exercise* | 0.42 (SD=0.49) |
| *Self-rated physical health (1-5)* | 3.22 (SD=1.08) |

Table S2 Basic regression, robustness test and heterogeneity test (China. 2018)

| Variables | Model 1:  Mental health | Model 2:  Mental health | Model 3:  Gini$\leq$0.4 | Model 4:  Gin$i>$0.4 | Model 5:  Education$\leq$2 | Model 6:  Education$>$2 |  |
| --- | --- | --- | --- | --- | --- | --- | --- |
|  |  |  | Mental health | | Mental health | |  |
|  | β (S.E.) | β (S.E.) | β (S.E.) | β (S.E.) | β (S.E.) | β (S.E.) |  |
| *Economic investment* | $-0.012$^**^  (0.005) | $-0.038$^**^  (0.017) | $-0.010$  (0.007) | $-0.013$^*^  (0.008) | $-0.010$^*^  (0.006) | $-0.027$^**^  (0.013) |  |
| *Emotional investment* | $-0.036$^**^  (0.019) | $-0.113$^*^  (0.060) | $-0.051$^**^  (0.023) | $-0.013$  (0.030) | $-0.029$  (0.023) | $-0.043$  (0.032) |  |
| *Time investment* | $-0.002$^*^  (0.001) | $-0.007$^*^  (0.004) | $-0.003$^*^  (0.002) | $-0.001$  (0.002) | $-0.002$  (0.002) | $-0.006$^**^  (0.003) |  |
| *Gini coefficient* | $-0.627$^**^  (0.308) | $-1.917$^*^  (0.984) | $-4.235$^***^  (1.000) | $-1.708$^***^  (0.625) | $-0.561$  (0.377) | $-1.091$^**^  (0.530) |  |
| *Age* | $0.002$  (0.002) | $0.007$  (0.005) | $0.002$  (0.002) | $0.001$  (0.002) | $0.001$  (0.002) | $0.008$^**^  (0.003) |  |
| *Gender* | $0.062$^***^  (0.020) | $0.197$^***^  (0.063) | $0.054$^**^  (0.026) | $0.073$^**^  (0.030) | $0.069$^***^  (0.023) | $0.031$  (0.037) |  |
| *Marital status* | $0.085$^***^  (0.022) | $0.252$^***^  (0.069) | $0.089$^***^  (0.033) | $0.088$^***^  (0.030) | $0.087$^***^  (0.028) | $0.069$^**^  (0.031) |  |
| *Work status* | $-0.003$  (0.008) | $-0.010$  (0.026) | $-0.002$  (0.010) | $-0.013$  (0.013) | $-0.003$  (0.009) | $-0.009$  (0.020) |  |
| *Educational level* | $0.025$^***^  (0.008) | $0.069$^***^  (0.025) | $0.026$^**^  (0.010) | $0.016$  (0.012) | $0.042$^***^  (0.014) | $-0.010$  (0.024) |  |
| *Social capital* | $0.005$  (0.005) | $0.015$  (0.016) | $0.002$  (0.007) | $0.005$  (0.008) | $0.004$  (0.006) | $0.009$  (0.010) |  |
| *Community trust* | $0.048$^***^  (0.005) | $0.150$^***^  (0.016) | $0.045$^***^  (0.007) | $0.053$^***^  (0.008) | $0.044$^***^  (0.006) | $0.062$^***^  (0.011) |  |
| *Net asset* | $0.009$^***^  (0.003) | $0.028$^***^  (0.010) | $0.011$^***^  (0.004) | $0.005$  (0.005) | $0.012$^***^  (0.004) | $0.005$  (0.006) |  |
| *Physical exercise* | $0.034$^*^  (0.020) | $0.107$^*^  (0.064) | $0.028$  (0.026) | $0.046$  (0.031) | $0.022$  (0.024) | $0.058$  (0.037) |  |
| *Self-rated physical health* | $0.162$^***^  (0.009) | $0.516$^***^  (0.029) | $0.179$^***^  (0.012) | $0.140$^***^  (0.015) | $0.153$^***^  (0.010) | $0.193$^***^  (0.019) |  |
| *_cons* | $1.968$^***^  (0.193) | $4.233$^***^  (0.611) | $3.274$^***^  (0.436) | $2.572$^***^  (0.353) | $1.974$^***^  (0.232) | $2.050$^***^  (0.360) |  |
| N | 4078 | 4078 | 2318 | 1760 | 3055 | 1023 |  |
| R-sq | 0.125 | 0.122 | 0.145 | 0.114 | 0.118 | 0.158 |  |

Table S3 The regression result of family educational investment and parental mental health and the mediation effect of social integration (China. 2018)

| Variables | Economic investment | | | Emotional investment | | | Time investment | | |
| --- | --- | --- | --- | --- | --- | --- | --- | --- | --- |
|  | Model 7:  Mental health | Model 8:  Social integration | Model 9:  Mental health | Model 10:  Mental health | Model 11:  Social integration | Model 12:  Mental health | Model 13:  Mental health | Model 14:  Social integration | Model 15: Mental health |
|  | β (S.E.) | β (S.E.) | β (S.E.) | β (S.E.) | β (S.E.) | β (S.E.) | β (S.E.) | β (S.E.) | β (S.E.) |
| *Economic investment* | $-0.012$^**^  (0.005) | $-0.035$^**^  (0.017) | $-0.010$^*^  $(0.005)$ |  |  |  |  |  |  |
| *Emotional investment* |  |  |  | $-0.041$^**^  (0.018) | $-0.106$^*^  (0.056) | $-0.034$^*^  $(0.018)$ |  |  |  |
| *Time investment* |  |  |  |  |  |  | $-0.003$^**^  (0.001) | $-0.008$^*^  (0.004) | $-0.002$^*^  (0.001) |
| *Social integration* |  |  | $0.066$^***^  (0.005) |  |  | $0.066$^***^  (0.005) |  |  | $0.066$^***^  (0.005) |
| *Gini coefficient* | $-0.620$^**^  (0.307) | $-0.735$  (0.939) | $-0.572$^*^  $(0.300)$ | $-0.614$^**^  (0.308) | $-0.718$  (0.939) | $-0.567$^*^  $(0.301)$ | $-0.635$^**^  (0.308) | $-0.776$  (0.939) | $-0.584$^*^  (0.301) |
| *Age* | $0.003$^*^  (0.002) | $0.018$^***^  (0.005) | $0.001$  (0.002) | $0.002$  (0.002) | $0.018$^***^  (0.005) | $0.001$  (0.002) | $0.002$  (0.002) | $0.017$^***^  (0.005) | $0.001$  (0.002) |
| *Gender* | $0.062$^***^  (0.020) | $-0.193$^***^  (0.062) | $0.075$^***^  (0.019) | $0.060$^***^  (0.020) | $-0.197$^***^  (0.062) | $0.073$^***^  (0.022) | $0.060$^***^  (0.020) | $-0.198$^***^  (0.062) | $0.073$^***^  (0.019) |
| *Marital status* | $0.082$^***^  (0.022) | $0.160$^**^  (0.073) | $0.072$^***^  (0.022) | $0.084$^***^  (0.022) | $0.165$^**^  (0.073) | $0.073$^***^  (0.022) | $0.085$^***^  (0.022) | $0.167$^*^  (0.074) | $0.074$^***^  (0.022) |
| *Work status* | $-0.004$  (0.008) | $-0.139$^***^  (0.026) | $0.005$  (0.008) | $0.002$  (0.008) | $-0.123$^***^  (0.025) | $0.010$  (0.008) | $0.001$  (0.008) | $-0.125$^***^  (0.025) | $0.009$  (0.008) |
| *Educational level* | $0.021$^***^  (0.008) | $-0.042$^*^  (0.024) | $0.024$^***^  (0.008) | $0.027$^***^  (0.008) | $-0.025$  (0.024) | $0.029$^***^  (0.008) | $0.025$^***^  (0.008) | $-0.030$  (0.023) | $0.027$^***^  (0.007) |
| *Social capital* | $0.004$  (0.005) | $0.021$  (0.016) | $0.002$  (0.005) | $0.006$  (0.005) | $0.026$  (0.016) | $0.004$  (0.005) | $0.005$  (0.005) | $0.025$  (0.016) | $0.004$  (0.005) |
| *Community trust* | $0.047$^***^  (0.005) | $0.214$^***^  (0.018) | $0.033$^***^  (0.005) | $0.047$^***^  (0.005) | $0.214$^***^  (0.018) | $0.033$^***^  (0.005) | $0.047$^***^  (0.005) | $0.213$^***^  (0.018) | $0.033$^***^  (0.005) |
| *Net asset* | $0.009$^***^  (0.003) | $0.012$  (0.009) | $0.008$^**^  (0.003) | $0.010$^***^  (0.003) | $0.016$^*^  (0.009) | $0.009$^***^  (0.003) | $0.010$^***^  (0.003) | $0.015$  (0.009) | $0.009$^***^  (0.003) |
| *Physical exercise* | $0.031$  (0.020) | $0.272$^***^  (0.062) | $0.013$  (0.020) | $0.036$^*^  (0.020) | $0.286$^***^  (0.062) | $0.017$  (0.020) | $0.034$^*^  (0.020) | $0.281$^***^  (0.062) | $0.015$  (0.020) |
| *Self-rated physical health* | $0.162$^***^  (0.009) | $0.429$^***^  (0.031) | $0.134$^***^  (0.009) | $0.162$^***^  (0.009) | $0.431$^***^  (0.031) | $0.134$^***^  (0.009) | $0.163$^***^  (0.009) | $0.432$^***^  (0.031) | $0.134$^***^  (0.009) |
| *_cons* | $1.939$^***^  (0.192) | $7.447$^***^  (0.617) | $1.450$^***^  (0.191) | $1.860$^***^  (0.188) | $7.219$^***^  (0.611) | $1.385$^***^  (0.186) | $1.866$^***^  (0.188) | $7.237$^***^  (0.610) | $1.389$^***^  (0.187) |
| N | 4078 | 4078 | 4078 | 4078 | 4078 | 4078 | 4078 | 4078 | 4078 |
| R-sq | 0.123 | 0.123 | 0.161 | 0.123 | 0.123 | 0.161 | 0.123 | 0.123 | 0.161 |

Table S4 The regression result of moderation effects of [social](javascript:;) [participation](javascript:;) and workload on the relationship between family educational investment and parental mental health (China. 2018)

| Variables | Model 16: Mental health | Model 17: Mental health |
| --- | --- | --- |
|  | β (S.E.) | β (S.E.) |
| *Economic investment* | $-0.012$^**^(0.005) | $-0.024$^**^(0.011) |
| *Emotional investment* | $-0.036$^**^(0.019) | $0.035$(0.050) |
| *Time investment* | $-0.002$^*^(0.001) | $-0.006$(0.004) |
| *Economic investment*$\times$[*Social*](javascript:;) [*participation*](javascript:;) |  | $0.017$^*^ (0.010) |
| *Emotional investment*$\times$[*Social*](javascript:;) [*participation*](javascript:;) |  | $-0.047$(0.047) |
| *Time investment*$\times$[*Social*](javascript:;) [*participation*](javascript:;) |  | $0.003$(0.004) |
| *Economic investment*$\times$*Workload* |  | $-0.004$(0.007) |
| *Emotional investment*$\times$*Workload* |  | $-0.049$^*^(0.027) |
| *Time investment*$\times$*Workload* |  | $0.002$(0.003) |
| *Gini coefficient* | $-0.627$^**^(0.308) | $-0.617$^**^(0.308) |
| *Age* | $0.002$(0.002) | $0.002$(0.002) |
| *Gender* | $0.062$^***^(0.020) | $0.067$^***^(0.020) |
| *Marital status* | $0.084$^***^(0.022) | $0.082$^***^(0.022) |
| *Work status* | $-0.003$(0.008) | $0.002$(0.008) |
| *Educational level* | $0.025$^***^(0.008) | $0.021$^***^(0.008) |
| *Social capital* | $0.005$(0.005) | $0.005$(0.005) |
| *Community trust* | $0.048$^***^(0.005) | $0.048$^***^(0.005) |
| *Net asset* | $0.009$^***^(0.003) | $0.009$^***^(0.003) |
| *Physical exercise* | $0.033$^*^(0.020) | $0.029$(0.020) |
| *Self-rated physical health* | $0.162$^***^(0.009) | $0.162$^***^(0.009) |
| *_cons* | $1.968$^***^(0.193) | $1.985$^***^(0.193) |
| N | 4078 | 4078 |
| R-sq | 0.125 | 0.128 |
